# Supplementary material for: Activin promotes skin carcinogenesis by attraction and reprogramming of macrophages
Source: EMBO Mol Med. 2016 Dec 8;9(1):27–45. doi: 10.15252/emmm.201606493 (PMC5210090; doi:10.15252/emmm.201606493)
Supplement: Supplementary file 1 — Appendix [file EMMM-9-27-s001.pdf]

Activin promotes skin carcinogenesis by attraction and reprogramming of macrophages

Maria Antsiferova[1], Aleksandra Piwko-Czuchra[1], Michael Cangkrama[1], Mateusz Wietecha[1], Dilara Sahin[1], Katharina Birkner[1], Valerie C. Amann[2], Mitchell Levesque[2], Daniel Hohl[3], Reinhard Dummer[3], and Sabine Werner[1*]

Appendix: Supplementary Information

Appendix Tables S1 - S5

Appendix Figures S1 - S3

References

Appendix Table S1. Genes significantly upregulated (FDR≤0.05, $|log_2Ratio| \geq 1$,) in FACS-sorted skin macrophages in 2-3 genotype comparisons (wt/Act *vs* wt/wt, HPV8/Act *vs* HPV8/wt and HPV8/Act *vs* wt/wt), grouped in functional categories based on literature search.

Functional category	Gene name	References
Adhesion/migration	*Cx3cr1*	Goda *et al*, 2000
	F11r	Gupta *et al*, 2000
	Itgb5	de Nichilo and Yamada, 1996
	Mmp14	Hara *et al*, 2011
	Itgal	Henderson *et al*, 2001
	Hck	Roach *et al*, 1997<br>Cougoule *et al*, 2010
Cancer-associated	*Cx3cr1*	Schmall *et al*, 2015
	F11r	Pont *et al*, 2013
	Spp1	Lin *et al*, 2015
	Mmp14	Ager *et al*, 2015
	C3	Markiewski *et al*, 2008;<br>Nunez-Cruz *et al*, 2012
	Tgfbr1	Byrne *et al*, 2008
	Mmp12	Hofmann *et al*, 2005;<br>Qu *et al*, 2011
	Havcr2	Yan *et al*, 2015
Angiogenesis	*F11r*	Rabquer *et al* 2010
	Spp1	Kale, 2014
	Mmp14	Basile *et* al, 2007;<br>Littlepage *et al*, 2010
	C3	Nozaki *et al*, 2006;<br>Nunez-Cruz et al, 2012
Anti-microbial/effector function	*Ass1*	Qualls *et al*, 2012
	Tgtp2	MacMicking, 2004
	Stat1	Spath *et al*, 2009
	C3	Markiewski and Lambris, 2007
	Hck	Nelson *et* al, 2009
	Axl	Seitz *et al*, 2007
	Mmp12	Houghton *et al*, 2009;<br>Marchant *et al*, 2014;
Immunoregulatory	*Klra2*	Gays *et al*, 2006
	Lair1	Colonna *et al*, 1999

	C3	Carroll, 2004
	Clec12a	Lahoud *et al*, 2009
	Hck	English *et* al, 1993
	Ly6e	Xu *et al*, 2014
	Axl	Sharif *et al*, 2006
	Tgfbr1	Takaki *et al*, 2006
	Kcnn4	Kang *et al*, 2014
	Mmp12	Bellac *et al*, 2014
	Havcr2	Monney *et al*, 2002

FDR: false discovery rate

Appendix Table S2. Primers used for RT-PCR or qRT-PCR.

Target gene	Forward sequence (5'-3')	Reverse sequence (5'-3')
HPRT	tgacactggcaaaacaatgca	ggtccttttcaccagcaagct
*INHBA/Inhba**	ggagaacgggtatgtggaga	acaggtcactgccttccttg
Gapdh	tcgtggatctgacgtgccgcctg	caccaccctgttgctgtagccgtat
Fst	agggaaagtgtatcacaaagt	gagttgcaagatccagaatg
E6 of HPV8	gcaggactgttcagtaccgtgcaac	acggcctctcccacagcaatct
Rps29	ggtcaccagcagctctacg	gtccaacttaatgaagcctatgtcc
Cxcl10	gggtctgagtgggactcaagggat	ccatccatcgcagcaccggg
Ccl19	gtcggagcctcggcctctca	ccccagagttggggctggga
Ccl22	ccgcaagcctggcgttgttttg	ggcgtcgttggcaaggctct
Arg1	cttaaagaaacagagtatgacgtgag	ctggttgtcaggggagtgtt
Cx3cr1	cctgcctctgagaaatggag	atctctccagcccctgaaat
Spp1	tgcagttctcctggctgaat	ctttcaccgggagggagga
Ccl17	acagtcagaaacacgatggca	tgctcgagccaccaatgtag
Mpp12	aaagtggggctttaagggaa	gttgcccagttgcttctagc

* the same primers were for used for the detection of human *INHBA* and mouse *Inhba* transcripts.

Appendix Table S3. Antibodies used for immunostaining.

Antibody	Source	Catalog No.
**Primary**		
Anti-Activin	R&D Systems, Minneapolis, MN	AF338
Anti-BrdU-FITC	Roche, Rotkreuz, Switzerland	1202693
Anti-CD206	R&D Systems	AF2535
Anti-CD31-biotin	Biolegend, San Diego, CA	102504
Anti-CD68-biotin	Serotec, Raleigh, NC	MCA1957B
Anti-Foxp3-biotin	eBioscience, San Diego, CA	13-5773-80
Anti-Keratin14	BAbCo, Richmond, CA	PRB-155P
Anti-LYVE1	AngioBio, San Diego, CA	11-034
Anti-Osteopontin	R&D Systems, Franklin Lakes, NJ	AF808-SP
Anti-Panendothelial Cell Antigen (MECA-32)	BD Biosciences, San Jose, CA	553849
Anti-TcRβ	eBioscience	14-5961-82
Anti-TcRδ-FITC	BD Biosciences	553177
**Secondary**		
Anti-goat-Cy2	Jackson Laboratories, Bar Harbor, MN	705-225-003
Anti-goat-HRP	DAKO, Glostrup, Denmark	0449
Anti-hamster-Cy3	Jackson Lab.	127-165-160
Anti-rabbit-Cy2	Jackson Lab.	111-165-003
Anti-rat-Cy3	Jackson Lab.	712-165-150
Streptavidin-Cy3	BioLegend	405215

Appendix Table S4. Antibodies used for flow cytometry.

**Antibody**	**Source**	**Catalog No.**
Anti-CCR3-PE	R&D Systems	FAB729P
Anti-CD11b-BV711	BioLegend	101242
Anti-CD16/CD32	BD Biosciences	553141
Anti-CD25-APC	eBioscience	17-0251
Anti-CD3-APC	BD Biosciences	553066
Anti-CD3-PE	BD Biosciences	553064
Anti-CD3-PE-CF594	BD Biosciences	562332
Anti-CD4-FITC	eBioscience	11-0041
Anti-CD45-AlexaFluor 700	BioLegend	103128
Anti-CD45-APC	eBioscience	17-0451
Anti-CD45-PB	BioLegend	103126
Anti-CD45-PE-Cy7	BioLegend	103114
Anti-CD45R/B220-FITC	BD Biosciences	553088
Anti-CD49b-PE	BD Biosciences	553858
Anti-CD64-APC	BioLegend	139305
Anti-CD64-BV421	BioLegend	139309
Anti-CD69-PE	eBioscience	12-0691
Anti-CD8a-PE	eBioscience	12-0081
Anti-F4/80-AlexaFluor488	BioLegend	123120
Anti-F4/80-BV421	BioLegend	123132
Anti-F4/80-PE	eBioscience	12-4801
Anti-Ly6C-PerCP-Cy5.5	BioLegend	127616
Anti-Ly6G-FITC	BD Biosciences	551460
Anti-MerTK-PE	R&D Systems	FAB5912P
Anti-MHCII-BV510	BD Biosciences	107635
Anti-TcRβ-APC	eBioscience	17-5961
Anti-TcRβ-PE	eBioscience	12-5961
Anti-TcRδ-FITC	BD Biosciences	553177

Appendix Table S5. GEO datasets and sample accession numbers of the published microarrays that were used to generate gene sets for GSEA. Complete gene sets, mapped gene set sizes, ranked gene lists, and GSEA reports can be found in dataset EV4.

**Description**	**GEO Datasets & Samples**	**Number of Genes in Sets**
Murine mammary tumor-associated macrophages *versus* mammary tissue macrophages	Series: GSE56755 (Franklin *et al*, 2014)	Generated via GEO2Enrichr, Characteristic Direction method
	TAM ("tumor associated macrophages, CD11blo"): GSM1368200, GSM1368201, GSM1368202	Up in TAM: 238 top genes
	Control ("mammary tissue macrophages, CD11bhi"): GSM1368198, GSM1368199	Down in TAM: 262 bottom genes
Murine late-stage breast tumor-associated macrophages *versus* splenic tissue macrophages	Series: GSE18404 (Ojalvo *et al*, 2009)	Generated via GEO2R, Default method using Benjamini & Hochberg p-value adjustment
	TAM ("tumor-associated macrophages"): GSM458919, GSM458920, GSM458921, GSM458922	Up in TAM: 236 top genes
	Control ("splenic macrophages"): GSM458923, GSM458924, GSM458925, GSM458926	Down in TAM: 236 bottom genes
Murine bone marrow tumor-associated macrophages *versus* bone marrow tissue macrophages	Series: GSE57785 (Galletti *et al*, 2016)	Generated via GEO2Enrichr, Characteristic Direction method
	TAM ("Monocytes/macrophages With MEC1 21d"): GSM1388466, GSM1388467, GSM1388468	Up in TAM: 240 top genes
	Control ("Monocytes/macrophages No MEC1 21d"): GSM1388463, GSM1388464, GSM1388465	Down in TAM: 260 bottom genes
Human Actinic Keratosis (AK)	Series: GSE2503 (Nindl *et al*, 2006)	Generated via GEO2R, Default method using Benjamini

versus normal skin (NS)		& Hochberg p-value adjustment
	AK ("cDNA ak"): GSM47612, GSM47613, GSM47614, GSM47615	Up in AK:<br>234 top genes
	NS ("cDNA no"): GSM47616, GSM47617, GSM47618, GSM47619	Down in AK:<br>174 bottom genes
Human Actinic Keratosis (AK) *versus* normal skin (NS)	Series: GSE63107	Generated via GEO2R, Default method using Benjamini & Hochberg p-value adjustment
	AK ("involved skin-pretreatment"): GSM1541373, GSM1541378, GSM1541383, GSM1541388, GSM1541393, GSM1541398	Up in AK: 394 top genes
	NS ("uninvolved skin-pretreatment"): GSM1541371, GSM1541376, GSM1541381, GSM1541386, GSM1541391, GSM1541396	Down in AK: 61 bottom genes
Monocytes sorted from human peripheral blood, differentiated into macrophages and differentially activated *in vitro*	Series: GSE46903 (Xue *et al*, 2014)	Calculated from Table S1D:<br>IFNg/control > 1.20:<br>78 top genes<br>IL4/control > 1.20:<br>36 top genes<br>TNF/control > 1.20:<br>79 top genes<br>LPS/control > 1.20:<br>120 top genes
	Data taken directly from Supplementary Table S1:<br>IFNg: M_GMCSF_IFNγ_72h<br>IL4: M_GMCSF_IL4_72h<br>TNF: M_GMCSF_TNF_72h<br>LPS: M_GMCSF_sLPS_72h<br>Control:<br>M_GMCSF_baseline_72h	
Human macrophage *versus* monocyte, dendritic cell, T cell signatures	Series: GSE46903 (Xue *et al*, 2014)	Generated via GEO2R, Default method using Benjamini & Hochberg p-value adjustment
	Macrophage: GSM numbers associated with "M_GMCSF_baseline_0h"	Monocyte vs macrophage:<br>95 top genes<br>Dendritic cell vs macrophage:<br>210 top genes<br>T-cell vs macrophage:<br>163 top genes<br>Macrophages vs monocyte, dendritic cell, T-cell:
	Monocyte: GSM numbers associated with "Monocyte_CD14+"	
	Dendritic cell: GSM numbers associated with "DC_imm"	

	T cell: GSM numbers associated with “Tconv” and “Treg”	97 top genes (shared by at least two comparisons of macrophage vs other cell type)

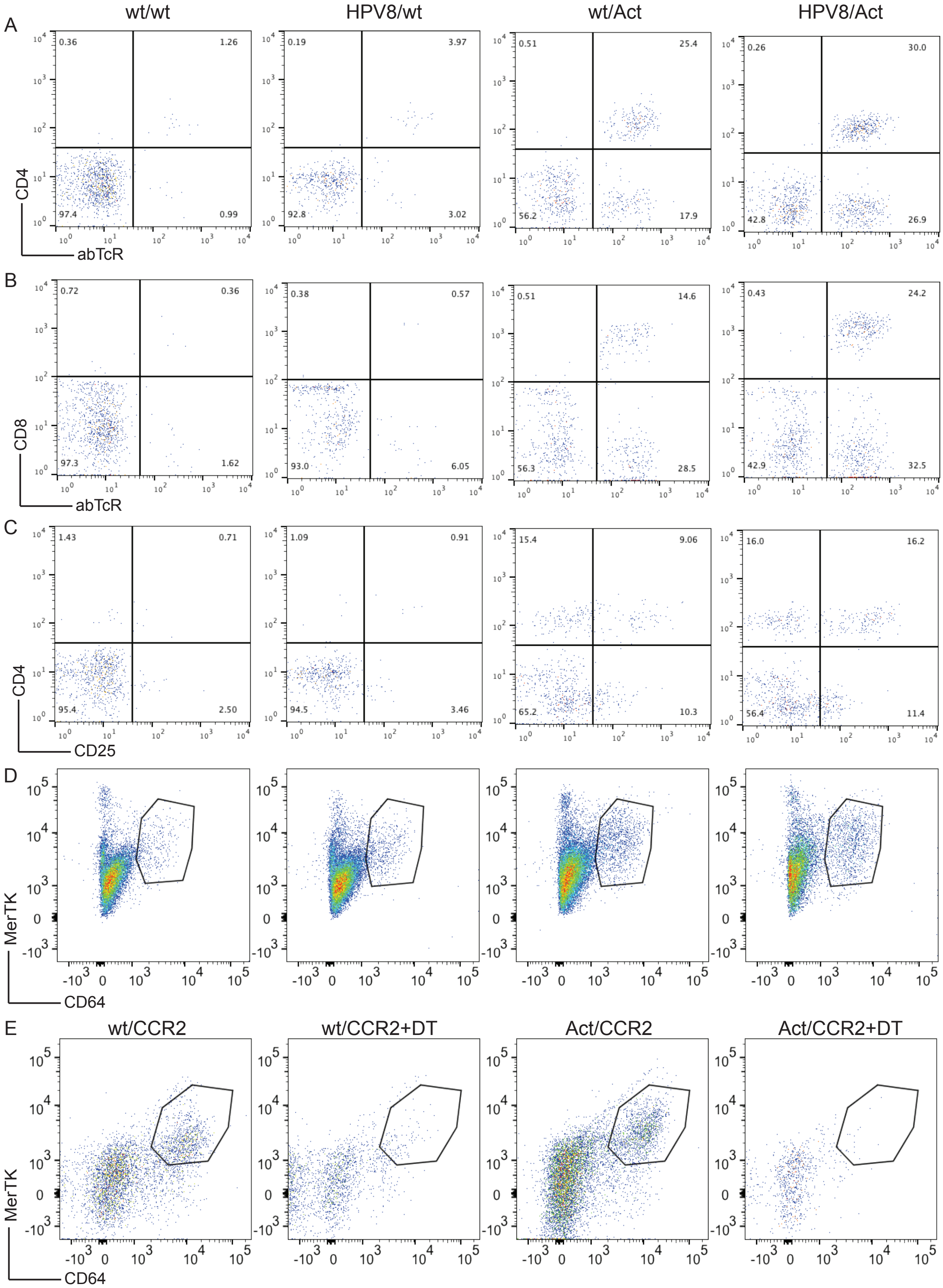

Appendix Fig S1. Original flow cytometry data. Representative flow cytometry scatter plots for quantified data shown in Fig 2D-F (A-C), Fig 3C (D), and Fig 3E (E).

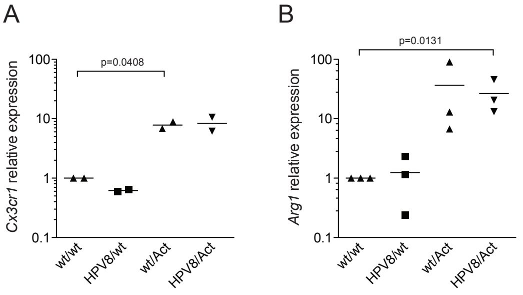

Appendix Fig S2. Activin induces expression of *Cx3c1* and *Arg1* in skin macrophages *in vivo*

(A, B) Expression of *Cx3cr1* (A) and *Arg1* (B) relative to *Rps29* was analyzed by qRT-PCR in F4/80$^+$CD11b$^+$ cells, FACS-sorted in 3 additional independent experiments from pools of 2-6 mice. Expression in one of the wt/wt samples was set to 1. N=2 for all groups in (A); N=3 for all groups in (B). One-sample t test was performed after log-transformation of expression data.

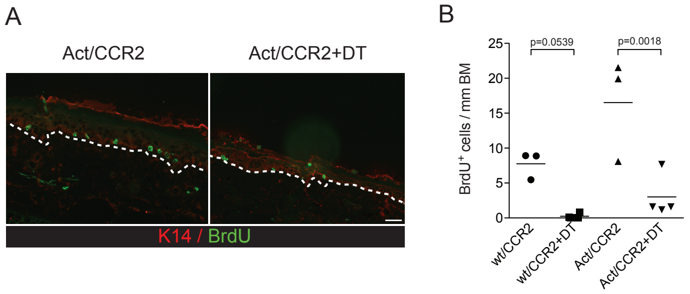

Appendix Fig S3. Macrophage depletion in Act/CCR2 mice reduces keratinocyte proliferation

(A) Representative pictures of anti-BrdU (green) and anti-K14 (red) stained cryosections of ear skin from 12 weeks old Act/CCR2 mice, untreated (control) or treated with diphtheria toxin (+DT) 3 times every 48h and analyzed 24h after the last injection. Scale bar: 50µm. Quantification of BrdU+ keratinocytes per mm of basement membrane is shown in (B). N=3 control mice; N=4 DT-injected mice. Statistical significance was performed using 1-way ANOVA and Bonferroni`s Multiple Comparison test.

References

Ager EI, Kozin SV, Kirkpatrick ND, Seano G, Kodack DP, Askoxylakis V, Huang Y, Goel S, Snuderl M, Muzikansky A, Finkelstein DM, Dransfield DT, Devy L, Boucher Y, Fukumura D, Jain RK (2015) Blockade of MMP14 activity in murine breast carcinomas: implications for macrophages, vessels, and radiotherapy. *J Natl Cancer Inst* 107: pii: djv017.

Basile JR, Holmbeck K, Bugge TH, Gutkind JS (2007) MT1-MMP controls tumor-induced angiogenesis through the release of semaphorin 4D. *J Biol Chem* 282: 6899-6905.

Bellac CL, Dufour A, Krisinger MJ, Loonchanta A, Starr AE, Auf dem Keller U, Lange PF, Goebeler V, Kappelhoff R, Butler GS, Burtnick LD, Conway EM, Roberts CR, Overall CM (2014) Macrophage matrix metalloproteinase-12 dampens inflammation and neutrophil influx in arthritis. *Cell Rep* 9: 618-632

Byrne SN, Knox MC, Halliday GM (2008) TGFbeta is responsible for skin tumour infiltration by macrophages enabling the tumours to escape immune destruction. *Immunol Cell Biol* 86: 92-97

Carroll MC (2004) The complement system in regulation of adaptive immunity. *Nat Immunol* 5: 981-986

Colonna M, Nakajima H, Navarro F, Lopez-Botet M (1999) A novel family of Ig-like receptors for HLA class I molecules that modulate function of lymphoid and myeloid cells. *J Leukoc Biol* 66: 375-381

Cougoule C, Le Cabec V, Poincloux R, Al Saati T, Mège JL, Tabouret G, Lowell CA, Laviolette-Malirat N, Maridonneau-Parini I (2010) Three-dimensional migration of macrophages requires Hck for podosome organization and extracellular matrix proteolysis. *Blood* 115: 1444-1452

De Nichilo MO, Yamada KM (1996) Integrin alpha v beta 5-dependent serine phosphorylation of paxillin in cultured human macrophages adherent to vitronectin. *J Biol Chem* 271: 11016-11022

English BK, Ihle JN, Myracle A, Yi T (1993) Hck tyrosine kinase activity modulates tumor necrosis factor production by murine macrophages. *J Exp Med* 178: 1017-1022

Franklin RA, Liao W, Sarkar A, Kim MV, Bivona MR, Liu K, Pamer EG, Li MO (2014) The cellular and molecular origin of tumor-associated macrophages. *Science* 344: 921-925

Fuentes-Duculan J, Suárez-Fariñas M, Zaba LC, Nograles KE, Pierson KC, Mitsui H, Pensabene CA, Kzhyshkowska J, Krueger JG, Lowes MA (2010) A subpopulation of CD163-positive macrophages is classically activated in psoriasis. *J Invest Dermatol* 131:391-400

Galletti G, Scielzo C, Barbaglio F, Rodriguez TV, Riba M, Lazarevic D, Cittaro D, Simonetti G, Ranghetti P, Scarfò L, Ponzoni M, Rocchi M, Corti A, Anselmo A, van

Rooijen N, Klein C, Ries CH, Ghia P, De Palma M, Caligaris-Cappio F, Bertilaccio MT (2016) Targeting Macrophages Sensitizes Chronic Lymphocytic Leukemia to Apoptosis and Inhibits Disease Progression. *Cell Rep* 14, 1748-1760

Gays F, Aust JG, Reid DM, Falconer J, Toyama-Sorimachi N, Taylor PR, Brooks CG (2006) Ly49B is expressed on multiple subpopulations of myeloid cells. *J Immunol* 177: 5840-5851

Goda S, Imai T, Yoshie O, Yoneda O, Inoue H, Nagano Y, Okazaki T, Imai H, Bloom ET, Domae N, Umehara H (2000) CX3C-chemokine, fractalkine-enhanced adhesion of THP-1 cells to endothelial cells through integrin-dependent and -independent mechanisms. *J Immunol* 164: 4313-4320

Gupta SK, Pillarisetti K, Ohlstein EH (2000) Platelet agonist F11 receptor is a member of the immunoglobulin superfamily and identical with junctional adhesion molecule (JAM): regulation of expression in human endothelial cells and macrophages. *IUBMB Life* 50: 51-56

Hara T, Mimura K, Seiki M, Sakamoto T (2011) Genetic dissection of proteolytic and non-proteolytic contributions of MT1-MMP to macrophage invasion. *Biochem Biophys Res Commun* 413: 277-281

Henderson RB, Lim LH, Tessier PA, Gavins FN, Mathies M, Perretti M, Hogg N (2001) The use of lymphocyte function-associated antigen (LFA)-1-deficient mice to determine the role of LFA-1, Mac-1, and alpha4 integrin in the inflammatory response of neutrophils. *J Exp Med* 194: 219-226

Hofmann HS, Hansen G, Richter G, Taege C, Simm A, Silber RE, Burdach S (2005) Matrix metalloproteinase-12 expression correlates with local recurrence and metastatic disease in non-small cell lung cancer patients. *Clin Cancer Res* 11: 1086-1092

Houghton AM, Hartzell WO, Robbins CS, Gomis-Ruth FX, Shapiro SD (2009) Macrophage elastase kills bacteria within murine macrophages. *Nature* 460: 637-641

Kang H, Kerloc'h A, Rotival M, Xu X, Zhang Q, D'Souza Z, Kim M, Scholz JC, Ko JH, Srivastava PK, Genzen JR, Cui W, Aitman TJ, Game L, Melvin JE, Hanidu A, Dimock J, Zheng J, Souza D, Behera AK, et al (2014) Kcnn4 is a regulator of macrophage multinucleation in bone homeostasis and inflammatory disease. *Cell Rep* 8: 1210-1224

Kale S, Raja R, Thorat D, Soundararajan G, Patil TV, Kundu GC (2014) Osteopontin signaling upregulates cyclooxygenase-2 expression in tumor-associated macrophages leading to enhanced angiogenesis and melanoma growth via alpha9beta1 integrin. *Oncogene* 33: 2295-2306

Lahoud MH, Proietto AI, Ahmet F, Kitsoulis S, Eidsmo L, Wu L, Sathe P, Pietersz S, Chang HW, Walker ID, Maraskovsky E, Braley H, Lew AM, Wright MD, Heath WR, Shortman K, Caminschi I (2009) The C-type lectin Clec12A present on mouse and human dendritic cells can serve as a target for antigen delivery and enhancement of antibody responses. *J Immunol* 182: 7587-7594

Lin CN, Wang CJ, Chao YJ, Lai, MD, Shan YS (2015) The significance of the co-existence of osteopontin and tumor-associated macrophages in gastric cancer progression. *BMC cancer* 15: 128

Littlepage LE1, Sternlicht MD, Rougier N, Phillips J, Gallo E, Yu Y, Williams K, Brenot A, Gordon JI, Werb Z (2010) Matrix metalloproteinases contribute distinct roles in neuroendocrine prostate carcinogenesis, metastasis, and angiogenesis progression. *Cancer Res* 70: 2224-2234

MacMicking JD (2004) IFN-inducible GTPases and immunity to intracellular pathogens. *Trends Immunol* 25: 601-609

Marchant DJ, Bellac CL, Moraes TJ, Wadsworth SJ, Dufour A, Butler GS, Bilawchuk LM, Hendry RG, Robertson AG, Cheung CT, Ng J, Ang L, Luo Z, Heilbron K, Norris MJ, Duan W, Bucyk T, Karpov A, Devel L, Georgiadis D, Hegele RG, Luo H, Granville DJ, Dive V, McManus BM, Overall CM (2014) A new transcriptional role for matrix metalloproteinase-12 in antiviral immunity. *Nat Med* 20: 493-502

Markiewski MM, Lambris JD (2007) The role of complement in inflammatory diseases from behind the scenes into the spotlight. *Am J Pathol* 171: 715-727

Markiewski MM, DeAngelis RA, Benencia F, Ricklin-Lichtsteiner SK, Koutoulaki A, Gerard C, Coukos (2008) Modulation of the antitumor immune response by complement. *Nat Immunol* 9: 1225-1235

Monney L, Sabatos CA, Gaglia JL, Ryu A, Waldner H, Chernova T, Manning S, Greenfield EA, Coyle AJ, Sobel RA, Freeman GJ, Kuchroo VK. (2002) Th1-specific cell surface protein Tim-3 regulates macrophage activation and severity of an autoimmune disease. *Nature* 415: 536-541

Nelson MP, Metz AE, Li S, Lowell CA, Steele C. (2009) The absence of Hck, Fgr, and Lyn tyrosine kinases augments lung innate immune responses to Pneumocystis murina. *Infect Immun* 77: 1790-1797

Nozaki M, Raisler BJ, Sakurai E, Sarma JV, Barnum SR, Lambris JD, Chen Y, Zhang K, Ambati BK, Baffi JZ, Ambati J. (2006) Drusen complement components C3a and C5a promote choroidal neovascularization. *Proc Natl Acad Sci U S A* 103: 2328-2333

Nunez-Cruz S1, Gimotty PA, Guerra MW, Connolly DC, Wu YQ, DeAngelis RA, Lambris JD, Coukos G, Scholler N (2012) Genetic and pharmacologic inhibition of complement impairs endothelial cell function and ablates ovarian cancer neovascularization. *Neoplasia* 14: 994-1004

Ojalvo LS, King W, Cox D, Pollard JW (2009) High-density gene expression analysis of tumor-associated macrophages from mouse mammary tumors. *Am J Pathol* 174, 1048-1064

Pong WW, Walker J, Wylie T, Magrini V, Luo J, Emnett RJ, Choi J, Cooper ML, Griffith M, Griffith OL, Rubin JB, Fuller GN, Piwnica-Worms D, Feng X, Hambardzumyan D, DiPersio JF, Mardis ER, Gutmann DH. (2013) F11R is a novel monocyte prognostic biomarker for malignant glioma. *PLoS One* 8: e77571

Qualls JE, Subramanian C, Rafi W, Smith AM, Balouzian L, DeFreitas AA, Shirey KA, Reutterer B, Kernbauer E, Stockinger S, Decker T, Miyairi I, Vogel SN, Salgame P, Rock CO, Murray PJ (2012) Sustained generation of nitric oxide and control of mycobacterial infection requires argininosuccinate synthase 1. *Cell Host Microbe* 12: 313-323

Qu P, Yan C, Du H (2011) Matrix metalloproteinase 12 overexpression in myeloid lineage cells plays a key role in modulating myelopoiesis, immune suppression, and lung tumorigenesis. *Blood* 117: 4476-4489

Rabquer BJ, Amin MA, Teegala N, Shaheen MK, Tsou PS, Ruth JH, Lesch CA, Imhof BA, Koch AE (2010) Junctional adhesion molecule-C is a soluble mediator of angiogenesis. *J Immunol* 185, 1777-1785

Roach T, Slater S, Koval M, White L, Cahir McFarland ED, Okumura M, Thomas M, Brown E. (1997) CD45 regulates Src family member kinase activity associated with macrophage integrin-mediated adhesion. *Curr Biol* 7: 408-417

Schmall A, Al-Tamari HM, Herold S, Kampschulte M, Weigert A, Wietelmann A, Vipotnik N, Grimminger F, Seeger W, Pullamsetti SS, Savai R. (2015) Macrophage and cancer cell cross-talk via CCR2 and CX3CR1 is a fundamental mechanism driving lung cancer. *Am J Respir Crit Care Med* 191: 437-447

Seitz HM, Camenisch TD, Lemke G, Earp HS, Matsushima GK (2007) Macrophages and dendritic cells use different Axl/Mertk/Tyro3 receptors in clearance of apoptotic cells. *J Immunol* 178: 5635-5642

Sharif MN, Sosic D, Rothlin CV, Kelly E, Lemke G, Olson EN, Ivashkiv LB. (2006) Twist mediates suppression of inflammation by type I IFNs and Axl. *J Exp Med* 203: 1891-1901

Spath GF, Schlesinger P, Schreiber R, Beverley SM (2009) A novel role for Stat1 in phagosome acidification and natural host resistance to intracellular infection by Leishmania major. *PLoS Pathog* 5: e1000381

Takaki H, Minoda Y, Koga K, Takaesu G, Yoshimura A, Kobayashi T (2006) TGF-beta1 suppresses IFN-gamma-induced NO production in macrophages by suppressing STAT1 activation and accelerating iNOS protein degradation. *Genes Cells* 11: 871-882

Xu X, Qiu C, Zhu L, Huang J, Li L, Fu W, Zhang L, Wei J, Wang Y, Geng Y, Zhang X, Qiao W, Xu J (2014) IFN-stimulated gene LY6E in monocytes regulates the CD14/TLR4 pathway but inadequately restrains the hyperactivation of monocytes during chronic HIV-1 infection. *J Immunol* 193: 4125-4136

Xue J, Schmidt SV, Sander J, Draffehn A, Krebs W, Quester I, De Nardo D, Gohel TD, Emde M, Schmidleithner L et al (2014) Transcriptome-based network analysis reveals a spectrum model of human macrophage activation. *Immunity* 40**:** 274-288

Yan W, Liu X, Ma H, Zhang H, Song X, Gao L, Liang X, Ma C (2015) Tim-3 fosters HCC development by enhancing TGF-beta-mediated alternative activation of macrophages. *Gut* 64: 1593-1604
