# Supplementary material for: Activin promotes skin carcinogenesis by attraction and reprogramming of macrophages
Source: EMBO Mol Med. 2016 Dec 8;9(1):27–45. doi: 10.15252/emmm.201606493 (PMC5210090; doi:10.15252/emmm.201606493)
Supplement: Supplementary file 2 — Expanded View Figures PDF [file EMMM-9-27-s002.pdf]

Expanded View Figures

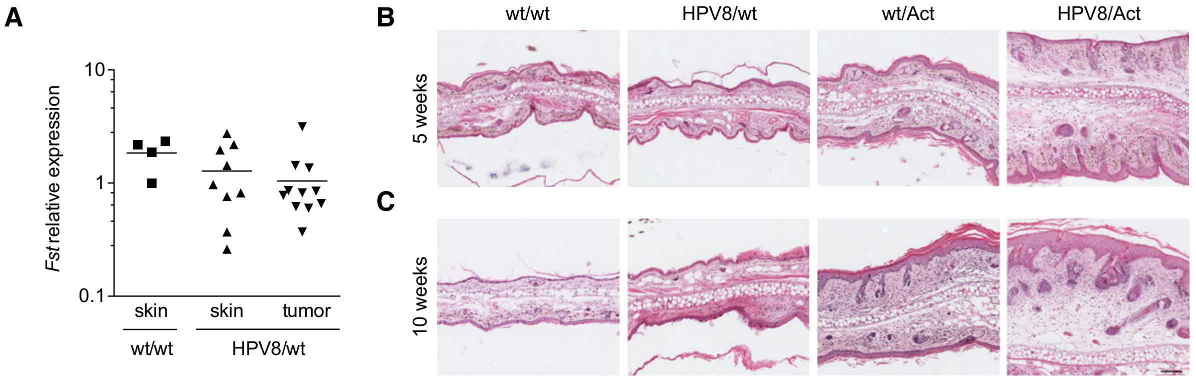

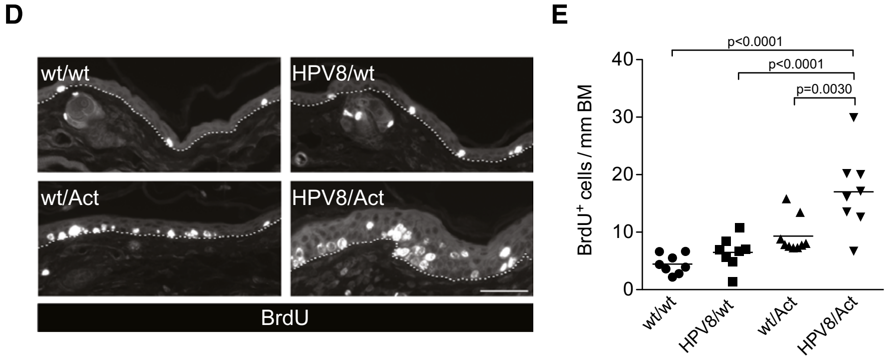

F

	HPV8/wt n	HPV8/wt %	HPV8/Act n	HPV8/Act %
back	7	35	14	70
ear	8	40	20	100
tail	0	0	7	35
eyelid	1	5	18	90
snout	2	10	17	85
whisker	5	25	7	35
anogenital	0	0	3	15
abdomen	2	10	2	10
head	1	5	1	5

G

	HPV8/wt n	HPV8/wt %	HPV8/Act n	HPV8/Act %
Acanthopapilloma	16	61.5	47	49
Trichoepithelioma - like skin lesion	0	0	6	6.2
Acanthopapilloma with trichoepitheliomatous differentiation	9	34.6	43	44.8
Sebaceous adenoma	1	3.9	0	0
Total	26		96	

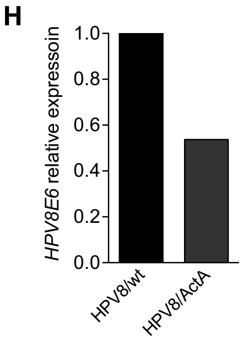

Figure EV1. Characterization of skin tumors in HPV8/wt and HPV8/Act mice.

A RNA samples from normal back skin of wt mice (N = 4, n = 4), non-affected back skin from HPV8/wt mice (N = 7, n = 9), and papillomas from HPV8/wt transgenic mice (N = 8, n = 11) were analyzed for the expression follistatin (*Fst*) relative to *Gapdh* by qRT–PCR. Expression level in one of the wild-type back skin samples was set arbitrarily to 1. N, number of mice; n, number of biopsies.

B, C Representative pictures of H&E-stained ear skin sections of 5 (B)- or 10 (C)-week-old mice. Scale bar: 100 μm.

D Representative pictures of BrdU-stained ear sections of 5-week-old mice. Dotted lines indicate the epidermal–dermal border. Scale bar: 50 μm.

E Quantification of BrdU-positive cells per mm of basement membrane (BM). N = 8 wt/wt, HPV8/wt, or HPV8/Act mice; N = 9 wt/Act mice. Statistical significance was determined using one-way ANOVA and Bonferroni's multiple comparisons test.

F Body sites where tumor development in HPV8/wt and HPV8/Act mice was observed; n, number of mice with tumors at the indicated body site; %, percentage of mice with at least one tumor at the respective body site, N = 20 mice per genotype.

G Histopathological analysis of tumors collected from HPV8/wt and HPV8/Act mice by a blinded histopathologist. n, number of tumors; %, percentage of tumors with the respective diagnosis among all tumors.

H RNA samples from MACS-sorted keratinocytes ($CD45^-$ epidermal fraction) were analyzed for the expression of the *HPV8E6* transgene relative to *Rps29* by qRT–PCR. Expression level in keratinocytes from HPV8/wt mice was set arbitrarily to 1. The experiment was performed with cells isolated and pooled from the ears of six mice per genotype.

 © 2016 The Authors

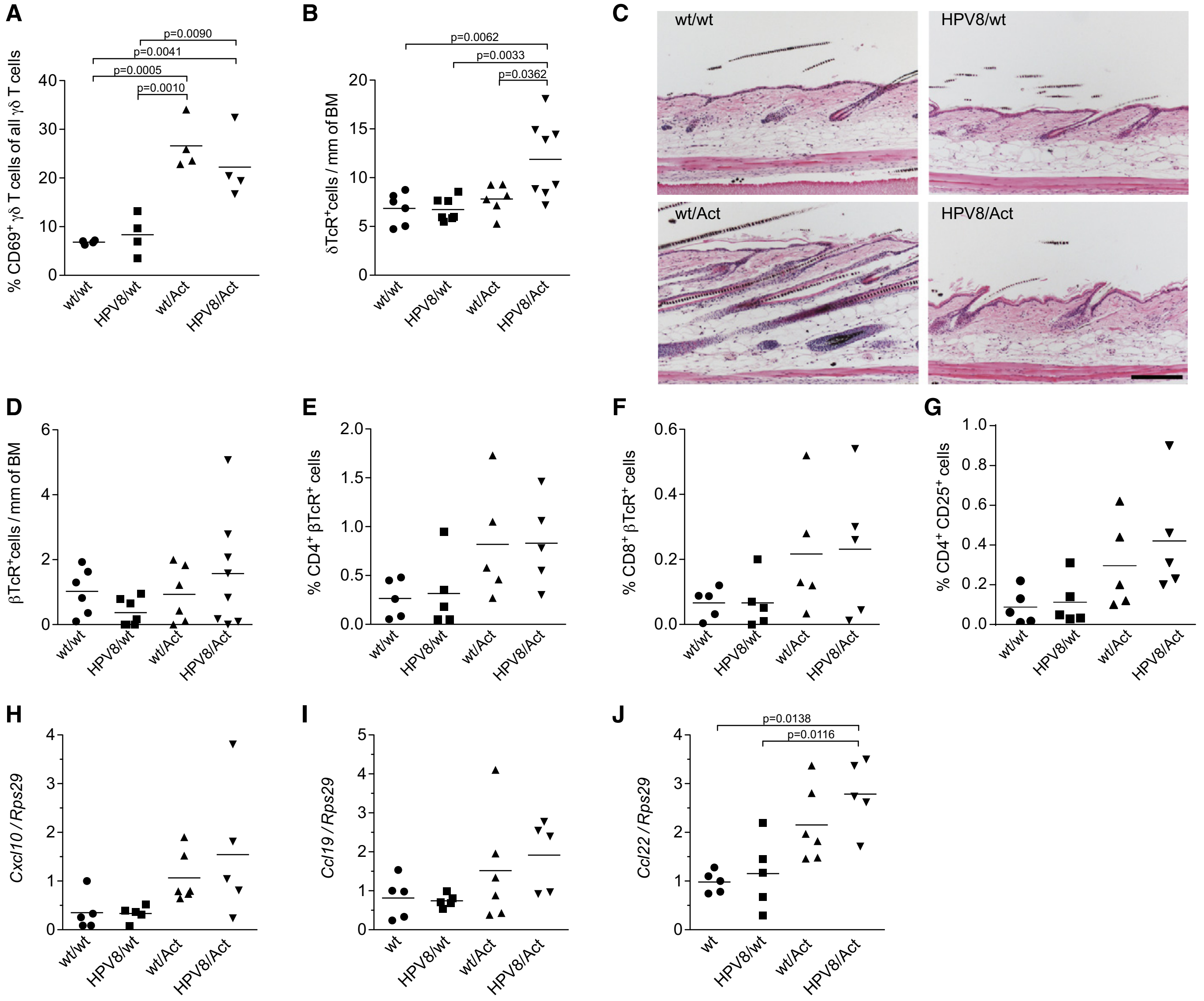

Figure EV2. Activin affects T cells and expression of T-cell chemokines in the skin.

- A Percentage of activated CD69^{+} γδ T cells in the ear skin epidermis of 14-week-old mice, analyzed by flow cytometry. wt/wt mice (N = 4); HPV8/wt mice (N = 4); wt/Act mice (N = 4); HPV8/Act mice (N = 4). N, number of mice.
- B Quantification of δTcR^{+} cells in cryosections of back skin from 10-week-old wt/wt mice (N = 6), HPV8/wt mice (N = 7), wt/Act mice (N = 6), and HPV8/Act mice (N = 8).
- C Representative pictures of H&E-stained sections of back skin at 10 weeks of age. Scale bar: 100 μm.
- D Quantification of βTcR^{+} cells in cryosections of back skin from 10-week-old wt/wt mice (N = 6), HPV8/wt mice (N = 7), wt/Act mice (N = 6), and HPV8/Act mice (N = 8).
- E–G Quantification of CD4^{+} (E), CD8^{+} (F), and CD4^{+}CD25^{+} (G) T cells in the ear skin dermis of 14-week-old mice by flow cytometry. Results from five experiments with ears pooled from at least three mice per genotype are shown.
- H–J RNA samples from total ear skin were analyzed for the expression of chemokines relative to *Rps29* by qRT–PCR. Expression in one of the wild-type ear skin samples was set to 1. Log$_2$-transformed data were used for statistical analysis. wt/wt mice (N = 5); HPV8/wt mice (N = 5); wt/Act mice (N = 6); HPV8/Act mice (N = 5).

Data information: Statistical significance was determined using one-way ANOVA and Bonferroni's multiple comparisons test of original (A–G) or log-transformed (H–J) data.

© 2016 The Authors

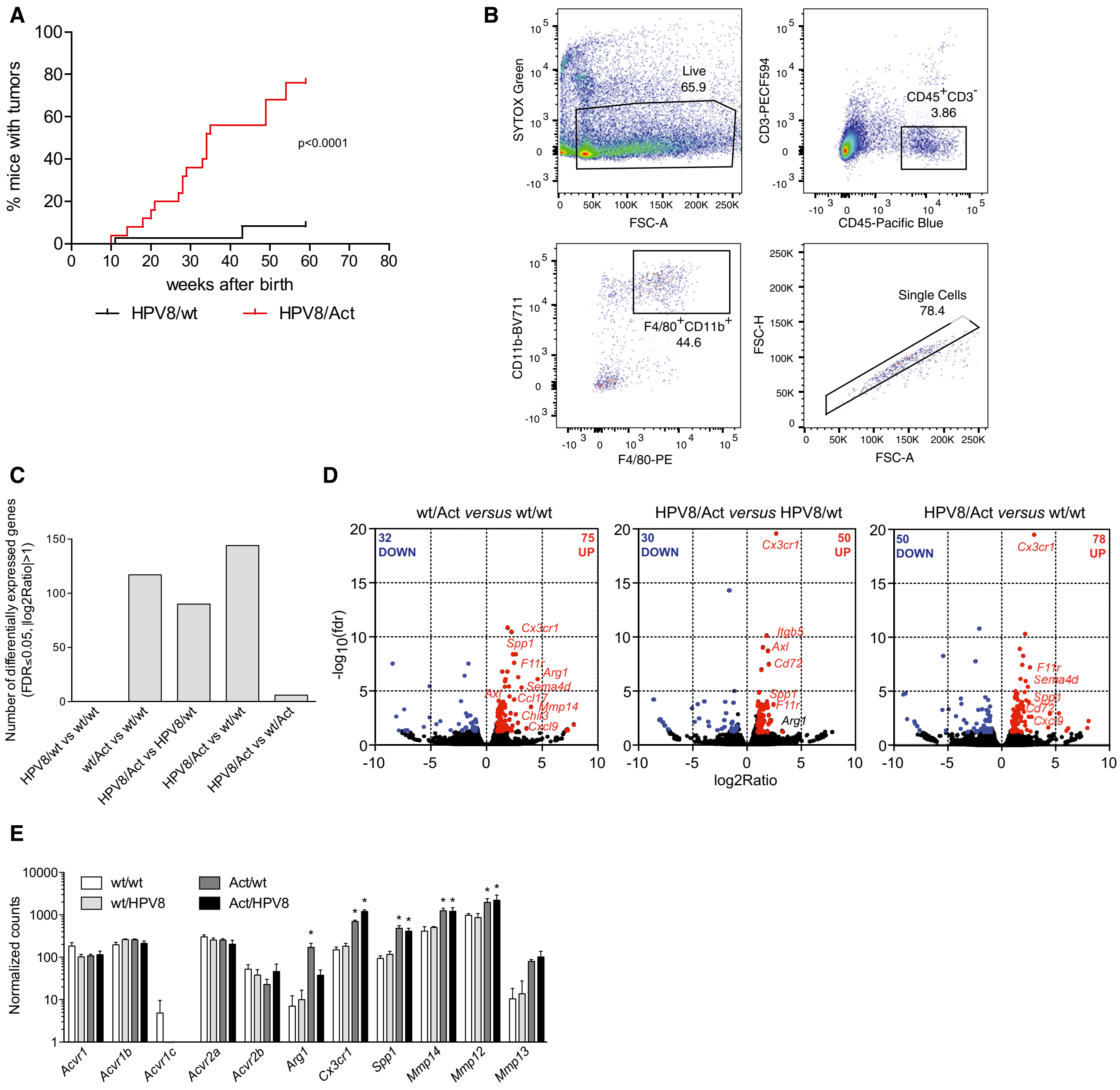

Figure EV3. Activin induces a pro-tumorigenic gene expression pattern in macrophages.

A Kinetics of tumor incidence in HPV8/wt and HPV8/Act mice after transfer of the mice into a new animal facility; N = 32 for HPV/wt, N = 23 for HPV8/Act mice. Statistical significance was determined using the log-rank (Mantel–Cox) test. N, number of mice.

B Gating strategy to enrich macrophages from ear skin of 13- to 15-week-old female mice by FACS. Representative pseudo-color plots of one of the wt/wt samples are shown. Single F4/80$^+$CD11b$^+$ cells were sorted after gating on single viable (SYTOX Green-negative) CD45$^+$CD3$^-$ cells.

C Number of differentially expressed genes (FDR < 0.05, $|\log_2 FC| > 1$) identified by RNA sequencing of F4/80$^+$CD11b$^+$ cells isolated from ear skin of 13- to 15-week-old female mice by FACS. Each replicate represents cells sorted from a pool of 3–6 mice per genotype in an independent experiment.

D Differential expression of genes was analyzed using the edgeR software package. Volcano plots show comparisons between wt/Act and wt/wt mice, HPV8/Act and HPV8/wt mice, or HPV8/Act and wt/wt mice. Significantly upregulated genes (FDR $\leq$ 0.05, $\log_2$ratio > 1) are highlighted in red, downregulated genes (FDR $\leq$ 0.05, $\log_2$ratio < −1) in blue.

E Normalized counts of transcripts for activin receptors and several tumor-promoting genes identified by RNA sequencing. N = 3. *FDR < 0.05 for comparison with wt/wt mice. Statistical significance was determined using exact test adapted for over-dispersed data. Exact P-values for different comparisons can be found in Dataset EV2. N = number of pools of 3–6 mice, mean ± SEM.

 © 2016 The Authors
