# Supplementary figures and images for: Activin promotes skin carcinogenesis by attraction and reprogramming of macrophages
Source: EMBO Mol Med. 2016 Dec 8;9(1):27–45. doi: 10.15252/emmm.201606493 (PMC5210090; doi:10.15252/emmm.201606493)

Original Blots

FIGURE 5C

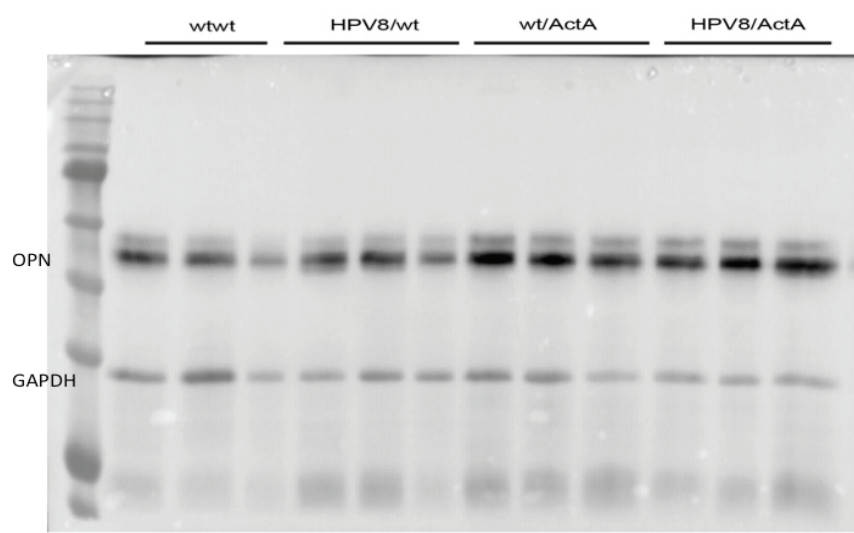

FIGURE 5H

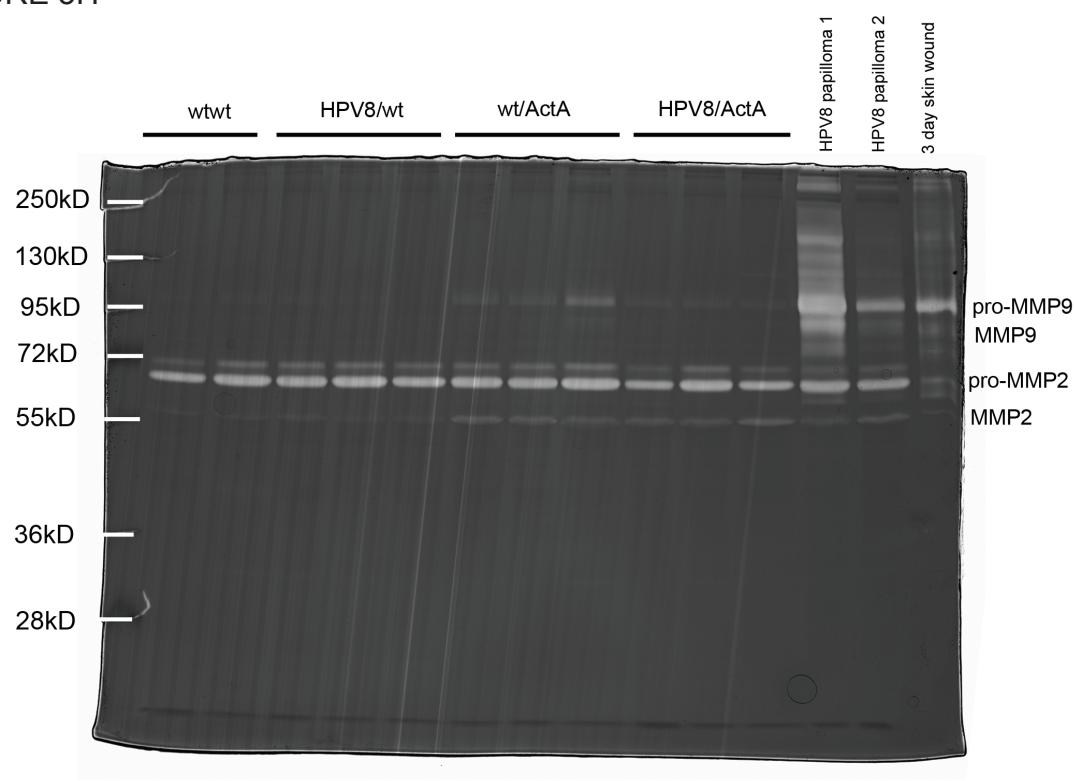

Supplement: Supplementary file 8 — Source Data for Figure 5 [file EMMM-9-27-s007.pdf]
